# Numerical Simulation of Hemodynamic Changes in Central Veins after Tunneled Cuffed Central Venous Catheter Placement in Patients under Hemodialysis

**DOI:** 10.1038/s41598-017-12456-7

**Published:** 2017-11-21

**Authors:** Liqing Peng, Yue Qiu, Zhongyi Huang, Chunchao Xia, Chenzhong Dai, Tinghui Zheng, Zhenlin Li

**Affiliations:** 1 Department of Radiology, West China Hospital, Sichuan University, Chengdu, 610041 China; 2 Department of Applied Mechanics, Sichuan University, Chengdu, 610065 China; 3 Vascular Surgery Department, West China Hospital, Sichuan University, Chengdu, 610041 China

## Abstract

The tunneled central venous catheter (CVC) plays an important role for hemodialysis patients, but CVC-related thrombosis in the central veins remain problematic. This study is the first try to numerically find out what hemodynamic parameters are predisposed to the initiation and formation of thrombus after CVC insertion. And the potential relationship between hemodynamic parameters and the incidence rates of thrombosis occurrence was explored. The results revealed that the CVC insertion led to a significant increase of hydraulic resistance, wide-ranging abnormally high wall shear stress (WSS), and a great loss of flow rotation in the vein. Moreover, the clinical data showed that thrombosis mainly occurred at sections where most blood flow lost spiral rotation after the CVC insertion, but no corresponding match was observed between the occurrence of thrombosis and the flow velocity or WSS. We speculate that the destruction of the flow rotation in the central vein is a precursor to the thrombus formation around CVC, and an introduction of spiral flow with the CVC insertion may possibly help to protect the central vein from thrombosis. Further animal and clinical experiments should be carried out to test and verify this speculation.

## Introduction

The tunneled central venous catheter (CVC) is a tube-like structure, which is placed into the central veins in the chest through the internal jugular, subclavian or femoral vein. CVC, arteriovenous (A-V) fistula and A-V graft are three main forms of vascular access to provide a life-line for patients with end stage renal diseases (ESRD)^1^. Although A-V fistula and A-V graft are preferential types of vascular access, tunneled CVC provides an important alternative for hemodialysis patients who have a poor vascular condition or who are waiting for an alternative access to mature^1,2^. Over the past 10 years, CVCs have played an increasing role in hemodialysis because they have advantages over arteriovenous access including the relative ease of placement, removal, replacement and the possibility for immediate use^1–4^.

A tunneled CVC may be used over a long period of time. However, complications including CVC-related bloodstream infection (also called catheter-related sepsis) and the thrombosis around CVC in the central veins remain problematic^5,6^. CVC-related bloodstream infections have an incidence of 1.1 to 5.5 episodes per 1000 catheter days and are associated with increased hospitalization, morbidity, and mortality^7^. However, CVC-related bloodstream incidence rate can be effectively reduced by several core interventions recommended by Centers for Disease Control and Prevention (CDC), including good hand hygiene practices, catheter care observations, education for both patients and staff on catheter care, chlorhexidine for skin antisepsis, catheter hub disinfection and antimicrobial ointment application during dressing change^7^. The clinical observation of a series of 120 patients under hemodialysis with tunneled CVC in the West China Hospital of Sichuan University showed that CVC dysfunction was mainly caused by thrombus formation around CVC with an incidence up to 53.3%. Several clinical studies also showed similar results^2,6,8–10^. Catheter-related thrombosis is a severe complication associated with the employment of CVC, which impairs the patency of central venous lumen and catheter performance and will finally result in catheter dysfunction^2,11^. Moreover, the falling of the thrombus may result in pulmonary embolism where the majority of associated deaths occur within hours^11^.

Risk factors for CVC-related thrombosis identified in the previous studies include the location of the catheter, long catheter remaining time, the degree of damaged endothelial cells, depth of the vascular injury, hypercoagulability, catheter diameter, repeated catheterization, diabetes, age older than 65 years, hypoalbuminemia, elevated lipoprotein-a level, lupus anticoagulant, female sex, elevated low-density lipoprotein cholesterol, alterations in genes regulating the coagulation cascade and hyperhomocysteinemia^2,8–12^. However, inconsistent or conflicting results were reported in these studies regarding the association between the risk factors and CVC-related thrombosis^2,9,13^. As a matter of fact, up to now, the definite mechanism and risk factors responsible for the initiation and progression of CVC-related thrombosis remain unknown, so there is still no effective way to reduce the risk of thrombosis for CVC use in clinical practice^2,14^.

It is well accepted that hemodynamics plays an important role in the initiation and progression of thrombosis in cardiovascular systems^13,15,16^. Wu *et al*.^15^ reported that thrombus accumulation was associated with decreased flow rate in continuous flow ventricular assist devices of Thoratec HeartMate II. Wen *et al*.^17^ found that the adoption of a helical bypass could help to prevent intimal hyperplasia and thrombosis at the distal anastomosis and improve the graft patency in artery bypass graft. Tian *et al*.^14^ reported that the insertion of a catheter into the coronary artery could cause marked increases of WSS and velocity in the left anterior descending (LAD) and increase the risk of platelet aggregation in LAD, which may make the coronary artery became more vulnerable to thrombus formation. The placement of CVC in the central veins follows a similar process to the insertion of a catheter into coronary arteries, and it is reasonable to speculate that the disturbed hemodynamics after the insertion of tunneled CVC may cause susceptible factors which can promote the thrombosis in ESRD patients. However, how the CVC insertion changes the hemodynamics in the central vein and whether hemodynamic parameters are closely related to thrombus formation after CVC insertion remain unknown. To the best knowledge of the authors, there has been no related investigation or report published.

Aiming to find out what undermines the formation of thrombus after CVC insertion in the central vein, a patient-specific model of a central vein with CVC was reconstructed based on computed tomography (CT) images, and the hemodynamic environment in the central veins before and after the placement of CVC were numerically simulated. The traditional hemodynamic parameters such as WSS and velocity vectors were computed and compared. And helicity, a good quantitative indicator of spiral flow was also investigated. Moreover, the incidence rates of thrombosis occurrence at different locations in the central vein were statistically presented based on the clinical data of a series of 120 patients in West China Hospital, and the statistical analysis of clinical data in patients was compared with the computed hemodynamic parameters, aiming at finding out which parameter is the most important indicator for the thrombosis after CVC insertion in the central vein.

## Results

**Figure 1:**
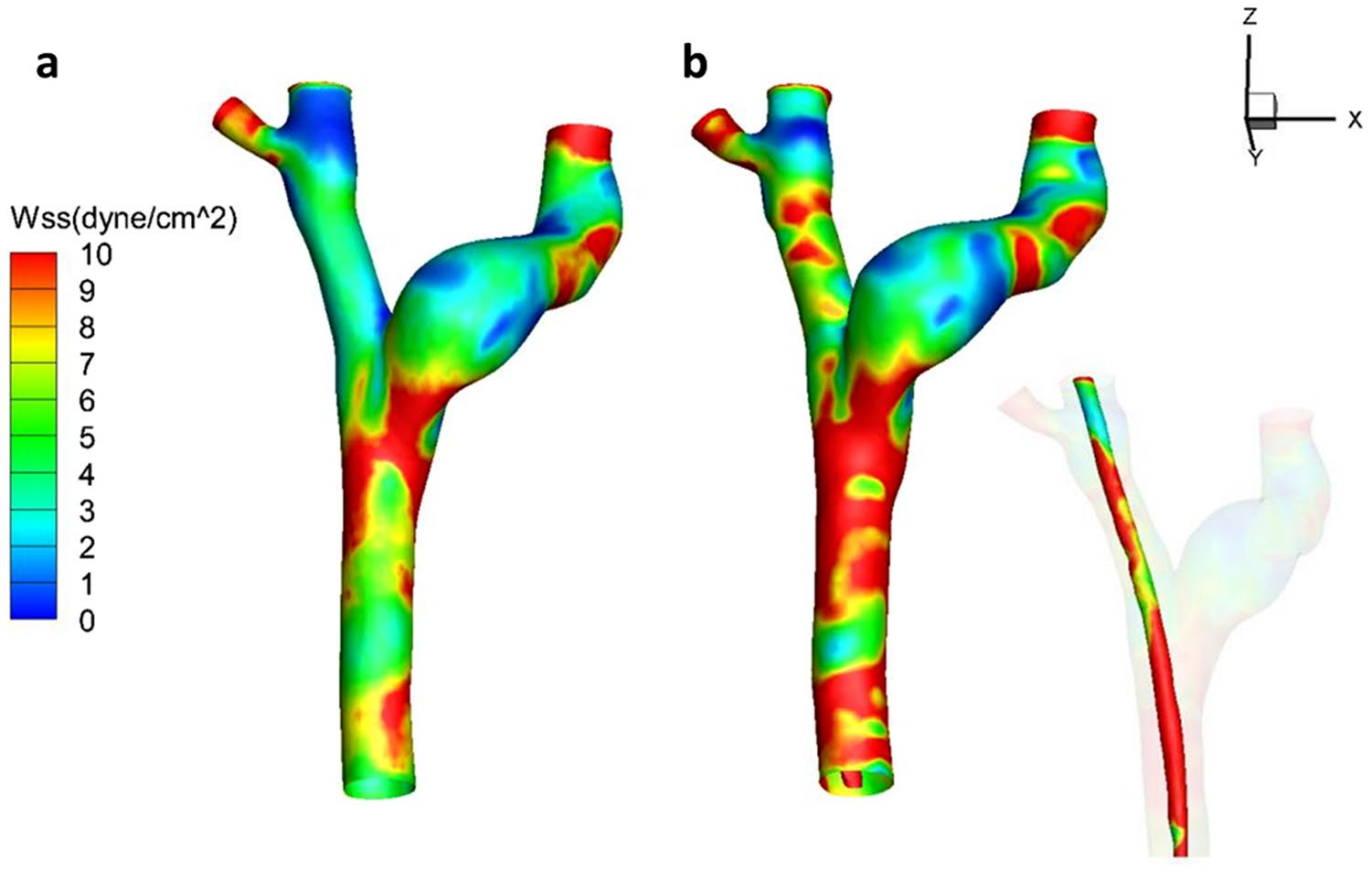
Contour maps of WSS distribution. (**a**) Central veins without CVC; (**b**) Central veins with CVC. (CVC = central venous catheter, WSS = wall shear stress).

The WSS contour maps of the vessel system are shown in Fig. 1. Physiological WSS values typically range from 1 to 10 dyne/cm^2^ in veins^18^. The low WSSs, smaller than 1 dyne/cm^2^, are marked in blue while high WSSs, larger than 10 dyne/cm^2^, are marked in red in Fig. 1. As is shown, the insertion of CVC didn’t make changes to the WSS distribution in the left brachiocephalic vein (LBV) but significantly expanded the region of high WSSs in other veins, especially in the superior vena cava (SVC). As a matter of fact, half of the right RBV, SVC and CVC tube wall were exposed to abnormally high WSS (larger than 10 dyne/cm^2^).

**Figure 2:**
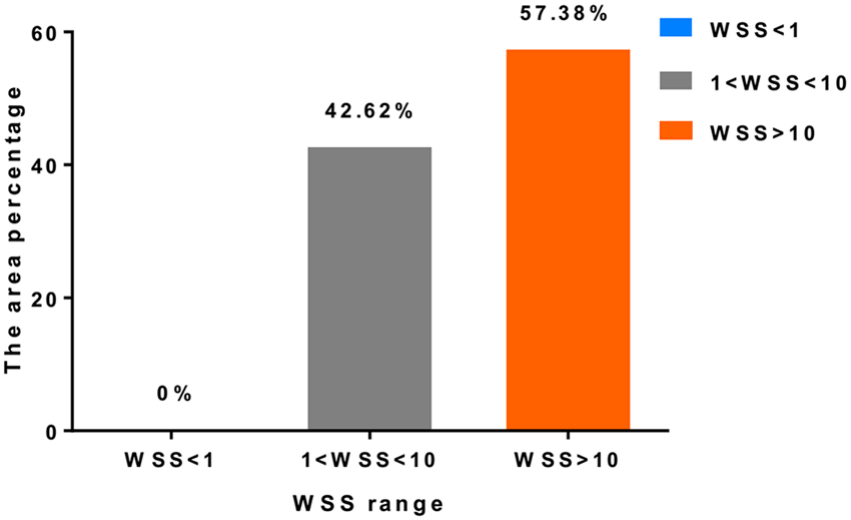
Histogram of the partition tables of WSS on the tube wall. (WSS = wall shear stress).

To have a more quantitative look at the WSS distribution on the tube, Fig. 2 presents the partition table of WSS. Again, no area of the tube was below the threshold value of 1 dyne/cm^2^, but up to 57.38% of the tube wall was covered with WSS values more than 10 dyne/cm^2^, and only 42.62% of the tube wall was in the normal WSS range.

**Figure 3:**
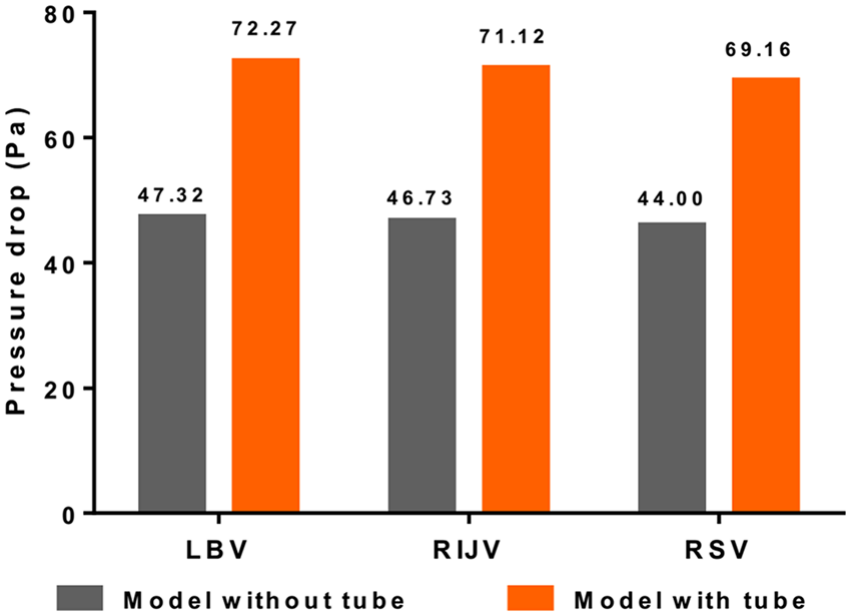
Histogram of the pressures drop from each inlet vein to the outlet of SVC with and without CVC. (SVC = superior vena cava, CVC = central venous catheter, RIJV = right internal jugular vein; RSV = right subclavian vein; LBV = left brachiocephalic vein).

Figure 3 presents the histogram of the pressure drop from each inlet to the outlet, with and without CVC. Obviously, because the CVC implantation greatly reduced the flow channel in the central vein, and the diameter reduction in the available channel reached 31.59%, a significant increase of the hydraulic resistance was raised by the introduction of the CVC. The pressure drop from the inlet (right internal jugular vein (RIJV), right subclavian vein (RSV), and LBV, respectively) to the outlet (SVC) increased from 47.32 Pa to 72.27 Pa, 46.73 to 71.12 Pa and 44.00 to 69.16 Pa before and after the CVC insertion, respectively. The increased percentages were 52.73%, 52.19% and 57.18%, respectively.

**Figure 4:**
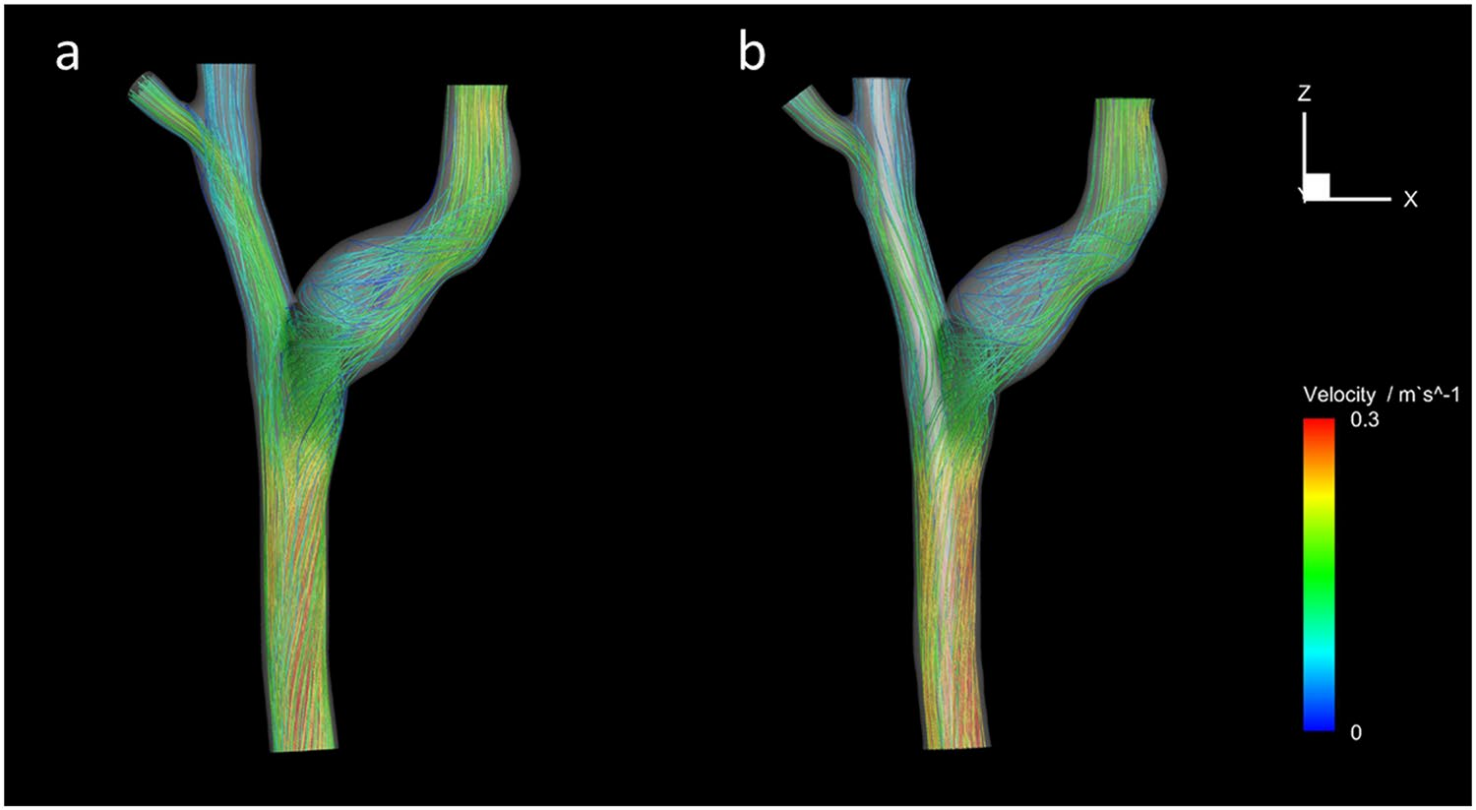
Flow streamlines inside the central veins and the velocity magnitude was differentiated using a color bar. (**a**) Central veins without CVC; (**b**) Central veins with CVC (Abbreviation as in Fig. 1).

Figure 4 presents the flow streamlines inside the central veins and the velocity magnitude is differentiated using a color bar, where the red represents high velocity while the blue indicates low velocity. It was shown that the flow remained laminar except at the bifurcation where flow was chaotic in the central vein without CVC. However, the insertion of CVC greatly disturbed the flow, the flow was sharply accelerated in the SVC and lost its laminar pattern at most parts in the vein system.

**Figure 5:**
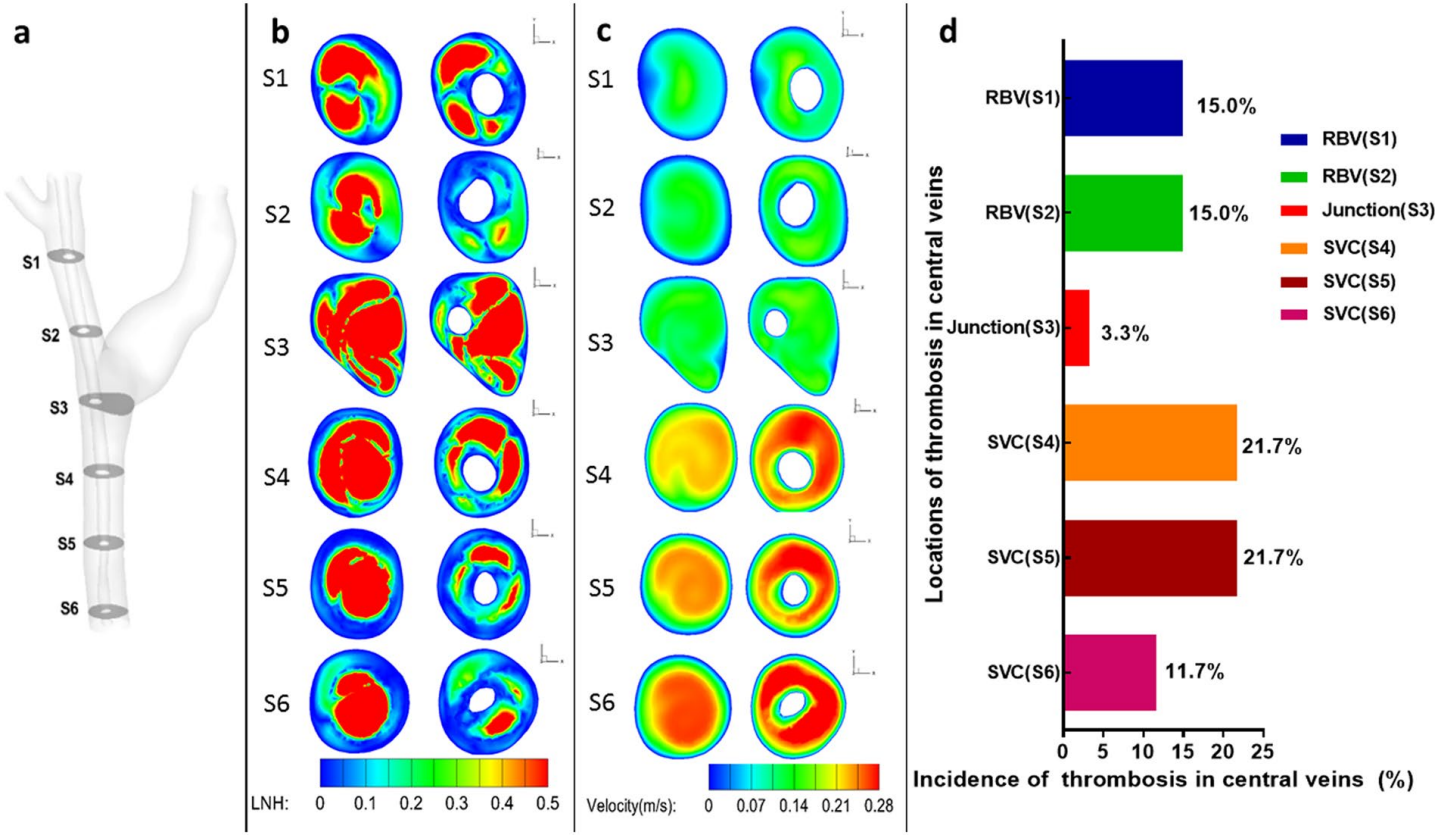
Common locations of CVC-related thrombosis in central veins in patients under hemodialysis. (**a**,**b**) Thrombosis (low density filling defect) around CVC (arrows) in right brachiocephalic vein (short arrow) and superior vena cava (long arrows) with more in superior vena cava; (**c**) Thrombosis in superior vena cava. (Abbreviation as in Fig. 1).

**Figure 6:**
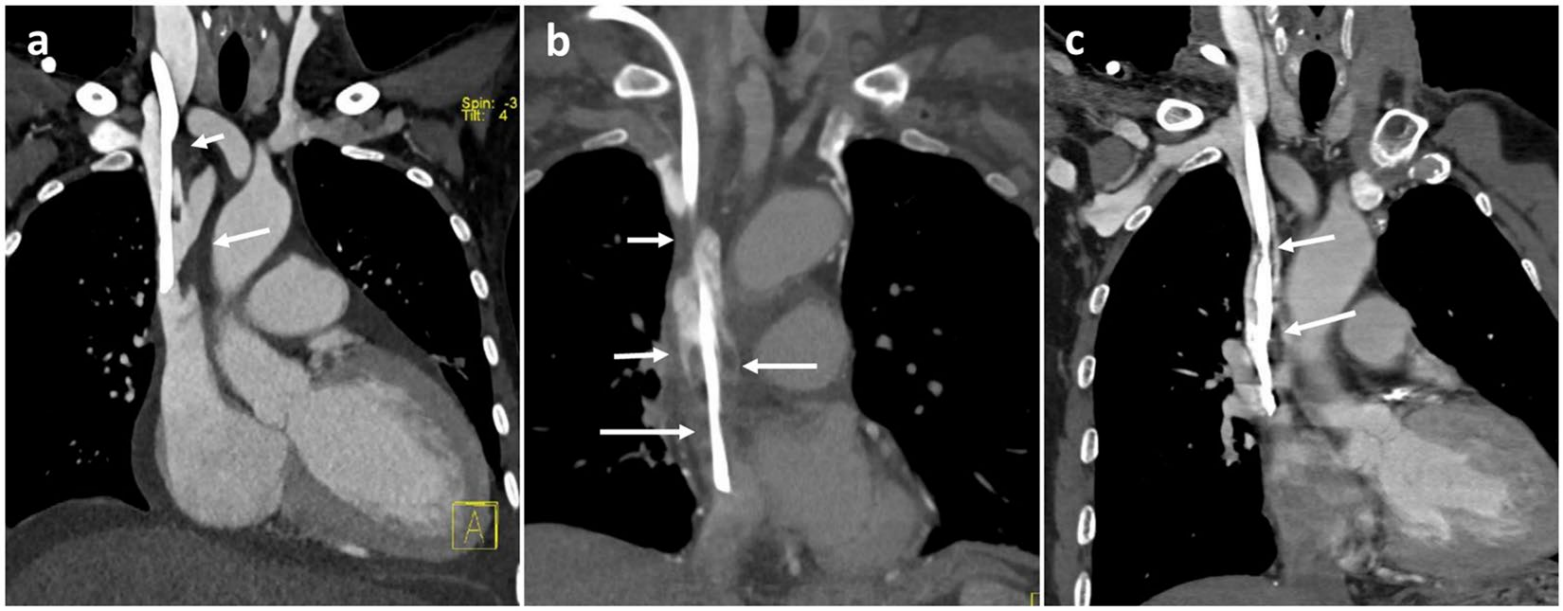
Comparison of the hemodynamic parameter in the indicated cross sections with the incidence rate of thrombosis. (RIJV = right internal jugular vein; RSV = right subclavian vein; RBV = right brachiocephalic vein; LBV = left brachiocephalic vein; Junction = the joint of right and left brachiocephalic vein; SVC = superior vena cava; CVC = central venous catheter).

The clinical observation showed that the thrombosis after CVC insertion in the central vein occurred mainly in the RBV and SVC (Fig. 5), and a comparison between the thrombosis distributions with the hemodynamic parameters was made in Fig. 6. Surprisingly, there was no sign that a large ratio of high WSS meant a high incidence rate of thrombosis. For example, cross section S2 had the smallest percentage of abnormally high WSS but the corresponding incidence rate of thrombosis was the second highest, and the section S4 was fully in the range of abnormally high WSS but had the same incidence rate as section S5 in which only 1/3 of the area was exposed to abnormally high WSS.

Recently, local normalized helicity (LNH), a nondimensional quantity which physically represents the angle between velocity and vorticity vectors, was introduced as a good quantitative indicator of the strength of helical flow^16^. A zero LNH indicates a purely axial or circumferential flow, whereas the modulus of LNH becomes 1 in a purely helical flow^16^. Figure 6b presents the contour map of LNH in the cross sections of the central veins. The low LNHs, smaller than 0.1, are marked in blue while high LNHs, larger than 0.5 are marked in red in the cross sections in Fig. 6b. Before the CVC insertion, the central zones of the cross sections were occupied by high LNH which indicates the flow is highly rotational there. But after the CVC insertion, except the cross section S3, due to the flow from the left brachiocephalic vein, the flow rotation remained, but other sections, especially S2, S6, the area with high LNH values significantly decreased and the LNHs in most area were close to zero, which means no spiral flow there.

Figure 6c pre*s*ents the contour map of the velocity magnitude at the indicated cross sections. The employment of the tube greatly increased the flow velocities in the SVC section but also resulted in the asymmetry of the flow distribution. Figure 6d is the histogram of the incidence rate of the thrombosis at different cross sections of the central veins, with the incidence rate being the statistical data based on a series of 120 patients undergoing hemodialysis with tunneled CVC. It shows that the incidence rate at section S1 (upper segment of brachiocephalic vein), S2 (lower segment of brachiocephalic vein), S3 (junction of LBV and RBV), S4 (upper segment of SVC), S5 (middle segment of SVC) and S6 (lower segment of SVC) were 15.0%, 15.0%, 3.3%, 21.7%, 21.7% and 11.7% respectively. The comparison of the incidence rates of thrombosis among different sections of central veins was statistically different (P < 0.05). In a word, the thrombosis after CVC insertion in the central veins occurred mainly in the RBV and SVC, and it is clear that the loss of the spiral motion (Cross section S1, S2, S4, S5, S6) implied a high incidence rate of thrombosis, and the least thrombosis incidence rate was observed at the junction (Cross section S3), which remained its high rotation, with and without CVC. However, no corresponding match was observed between the thrombosis occurrence and the flow velocity.

## Discussion

In recent years, tunneled CVCs have played an increasing role in hemodialysis because they have advantages over arteriovenous access including the relative ease of placement, removal and replacement and the possibility for immediate use^19^. Tunneled CVCs are meant to be in use for weeks to months, and possibly even years. Unfortunately, thrombosis is a common cause of early failure of CVCs^2,9,13^. Therefore, there is a clinical imperative to determine the risk factors for CVC-related thrombosis. Several previous studies have been conducted to determine the potential risk factors for CVC-related thrombosis, however, the conclusions are conflicting or inconsistent, and the main cause of thrombosis remains unknown^2,9,11^.

The current study is the first attempt to explore the underlying mechanism of thrombus formation after CVC insertion in the central veins of a hemodialysis patient with regard to hemodynamics. The results revealed that the CVC insertion significantly increased the flow resistance and asymmetry which resulted in a wide-ranged abnormal WSS, but decreased the flow rotation inside the central vein. And most of the flow in the RBV and SVC became purely axially directed after the CVC insertion. Moreover, the comparison between the computed hemodynamic parameters with the clinical data indicated that the CVC-related thrombosis was closely related to the flow rotation, but the relativity between the thrombosis occurrence and the flow velocity or the WSS were not found.

WSS is the frictional force of the blood flow that acts on the vessel wall, and can be sensed directly on an endothelial cell at the luminal surface^20^. It is fully believed that abnormal WSS is an important reason for the initiation and formation of thrombus. Platelet activation, a precursor for thrombosis, has been shown to be a function of elevated shear stress^21^. So, it is surprising that a close correlation between the incidence rate and abnormal WSS was not found in the current study. We speculate that one possible reason is that we have assumed an equal flow rate before and after the CVC insertion in the central vein, which may not match the reality. Actually, if the perfusion pressure of the organs remains unchanged before and after an operation, the increased hydraulic resistance will inevitably lead to a decrease of flowrate. So, on one hand, the percentage of abnormally high WSS after CVC insertion will dramatically decrease. But on the other hand, it will further decrease the velocity of slow moving blood in the vein system and part of the flow may become stagnant. Consequently, the blood velocity is highly susceptible where below the “thrombotic threshold velocity”, which leads to thrombus accumulation on the graft or vessel surface, and finally results in occlusion^22^. Moreover, because the stagnation flow zones are associated with very low shear conditions and some parts of the vessel wall will be exposed to abnormally low WSS that means the fluid shearing forces of the blood will not be sufficient to overwhelm the forces associated with cell–cell interactions. Whether the CVC insertion leads to abnormal low WSS and whether and how the thrombosis is related to the low WSS distribution is left to future research.

It is very interesting and inspiring to find that the CVC-related thrombosis mainly occurred at sections where the flow lost most of its spiral rotation. In arterial systems, spiral blood flow has been suggested as a normal physiological flow phenomenon to protect the vessel wall from damage by reduction of the laterally directed forces, to facilitate the blood flow transportation, to prevent the accumulation of atherogenic low density lipoproteins on the luminal surfaces of arteries, and reduce the adhesion of blood cells on the arterial surface which protect the arteries from the pathology of thrombosis^14,15^. According to our preliminary results, the loss or marked reduction of spiral flow in the central veins after CVC insertion might be risk factors for thrombosis for patients undergoing hemodialysis. Thus, we speculate that the presence of spiral blood flow in the central veins may play a similar role in protecting the veins from thrombosis as in the arteries, or it might be important to maintain the spiral flow in the central veins after CVC insertion to decrease the occurrence of thrombosis.

Geometric configuration is a decisive factor to the hemodynamics in the venous system. Different geometric factors including CVC shape, diameter and length will cause different disturbance to the flow in the central veins. Furthermore, the surgical planning of CVC insertion such as CVC location, CVC curvature and implantation depth for a specific patient may be varied. We expect that, in the near future, a patient-specific CFD study of the hemodynamics in the central veins after CVC insertion could be conducted to optimize the tube geometry and operation plan so as to reduce the disturbance to the blood flow to the greatest extent, which may help to improve the postoperative effect. Additionally, we believe our research might be used to help design new types of CVC, which may keep or introduce spiral flow to the central vein to inhibit thrombus formation^23^. However, whether this design conception can reduce the incidence of CVC-related thrombosis in hemodialysis patients should be further studied.

## Methods

### Model Geometry

This study was conducted in accordance with the principles of the Declaration of Helsinki and met the requirement of medical ethics. The Ethical Review Committee of the West China Hospital of Sichuan University (Chengdu, Sichuan, China) approved this research. As our study was purely observational and retrospective in nature and used anonymized data, patient approval and informed consent were waived.

**Figure 7:**
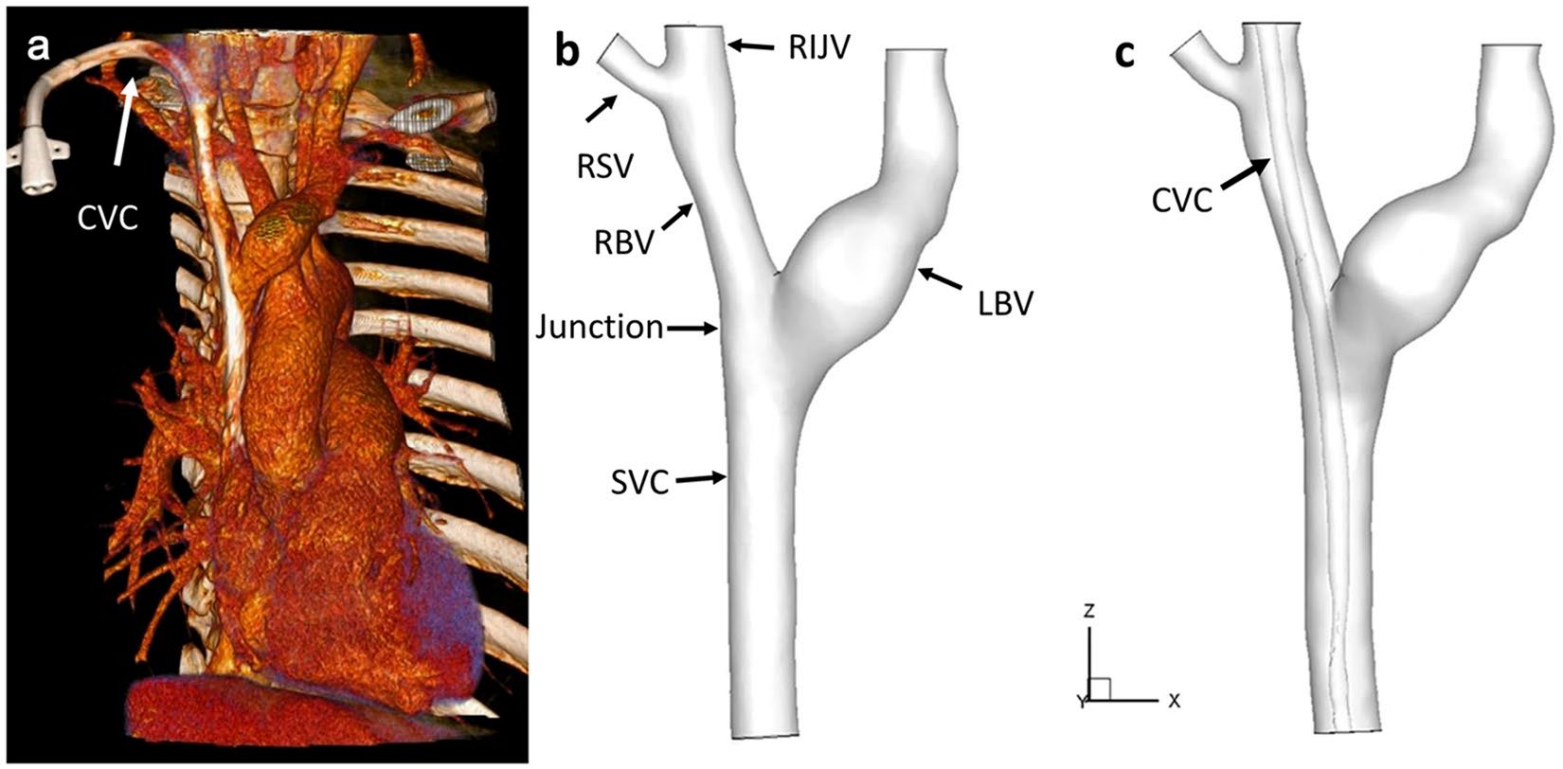
Typical CT image and 3D reconstruction of central veins with CVC inserted through right internal jugular vein. (**a**) 3D volume rendered image of central veins with thin-slice CT scan images; (**b**) 3D reconstruction of the central veins without CVC after segmentation using Mimics v10.0; (**c**) 3D reconstruction of the central veins with CVC. (Abbreviation as in Fig. 6).

Patient-specific data sets were provided by the West China Hospital of Sichuan University (Chengdu, Sichuan, China) and included computed tomography angiography (CTA) data. Thin-slice CTA data was obtained with a multi-slice computed tomography scanner (Somatom Definition Flash, Siemens Medical Solutions, Germany). CTA images of the central vein were obtained (Fig. 7a) and the three-dimensional geometry was then reconstructed from CTA images using the commercially available software Mimics (version 14.0; Materialise, Plymouth, Mich) (Fig. 7b,c). As shown, the CVC was inserted through the right internal jugular vein, and the geometrical parameters were measured at 5 days post-operatively. Vessel diameters were 5.8 mm, 3.6 mm, 5.5 mm and 6.4 mm for the RIJV, RBV, LBV and SVC, respectively.

### Governing Equations

In the current study, the blood was assumed to be an incompressible, laminar, steady, homogenous and Newtonian fluid, and the corresponding governing equations are given as

1 $$\rho (\vec{u}\cdot \nabla )\vec{u}+\nabla p-\mu {\rm{\Delta }}\vec{u}=0$$

2 $$\nabla \cdot \vec{u}=0$$

where $\vec{u}$ and *p* represent, respectively, the fluid velocity vector and the pressure. *ρ* and *μ* are the density of 1045 *g/m* ^3^ and dynamic viscosity of 3.5 × 10^−3^ *kg*/*m*·*s*.

### Boundary conditions

The patient-specific blood-flow information was obtained from 4D flow phase-contrast magnetic resonance imaging (PC-MRI) (Siemens, MAGNETOM Skyra, Germany) measurements of flow rates. The corresponding flow rates for the RIJV, RBV, LBV and SVC were 3.39 ml/sec, 8.76 ml/sec, 10.86 ml/sec, 28.43 ml/sec, respectively. To simplify the computation, some veins like the right external jugular vein, azygos vein, left internal and external jugular veins, left subclavian vein were occluded.

The graft and vessel wall were assumed to be rigid and nonslip as a preliminary study.

### Numerical simulation

**Figure 8:**
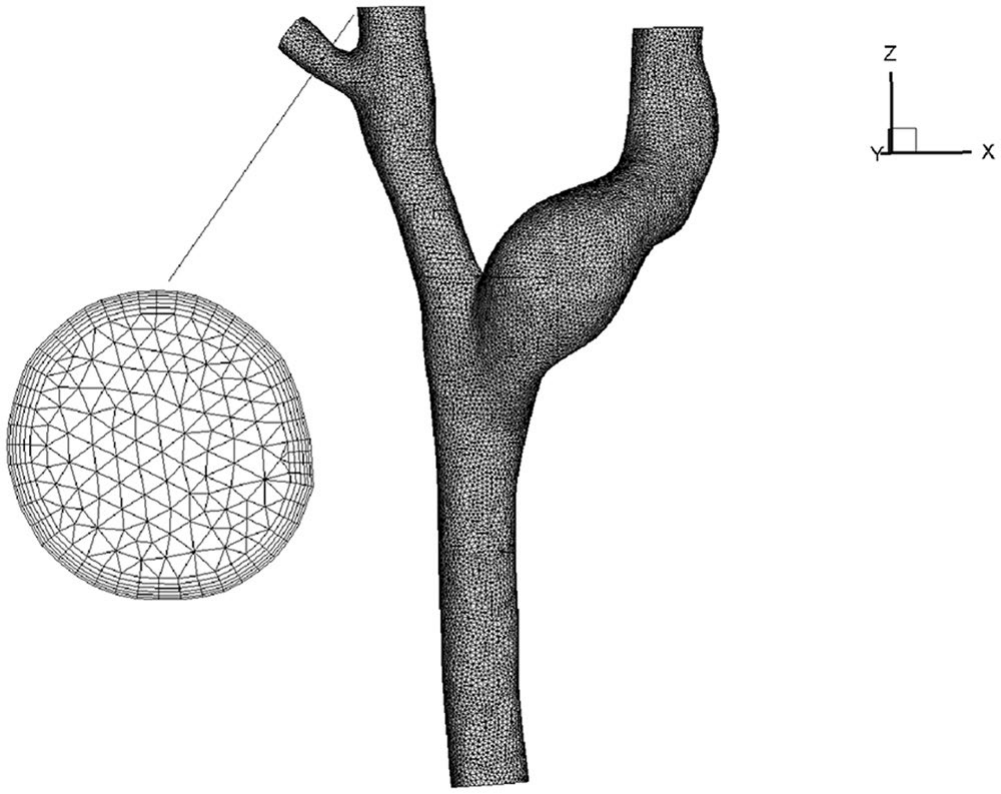
Sketch of mesh generation and the zoom-in view of the boundary layer mesh.

A finite volume mesh with 590086 or 315019 unstructured tetrahedral elements and 170521 or 103490 nodes were generated for the central venous system without or with CVC respectively, using an automatic mesh generator ICEM (ANSYS Inc., Pittsburgh, PA). The boundary layer was resolved by placing the first four grid nodes at approximately 15, 17, 19 and 21μm away from the wall, and we supplemented the zoom-in view of the boundary layer mesh as shown in Fig. 8.

The flow visualization and analysis were completed by the commercial CFD software Ansys FLUENT 16.0 which was based on the finite volume method. The default segregate implicit 3D solver was applied. Discretization of the equations involved a second order upwind differencing scheme, SIMPLE, that was adopted for the pressure velocity correction and the residual error convergence threshold was set as 1e-6.

### Statistical Analysis

As this is an observational and descriptive study. Continuous data are expressed as specific values. Categorical variables are presented as percentages. Descriptive statistics were calculated. The incidence rates of thrombosis in different locations of central veins in the series of 120 patients with thrombus in central veins were presented as constituent ratio. Comparisons between incidences of thrombosis in different locations of central veins were performed by Pearson χ^2^ test. A 2-tailed probability value of <0.05 indicated statistically significant. Statistical analysis was performed using commercially available software (SPSS for Windows, 11.5).
